# Glucocorticoid therapy does not delay viral clearance in COVID-19 patients

**DOI:** 10.1186/s13054-020-03287-6

**Published:** 2020-09-21

**Authors:** Jingjing Ji, Jinxia Zhang, Ziyun Shao, Qifeng Xie, Li Zhong, Zhifeng Liu

**Affiliations:** 1 Department of Critical Care Medicine, General Hospital of Southern Theater Command of PLA, Guangzhou, 510010 China; 2 Cardiovascular Department, General Hospital of Southern Theater Command of PLA, Guangzhou, 510010 China; 3 Department of Nephrology, General Hospital of Central Theater Command of PLA, Wuhan, 430070 China; 4 Department of Urology Surgery, General Hospital of Southern Theater Command of PLA, Guangzhou, 510010 China; 5 Department of Critical Care Medicine, The First Affiliated Hospital, Guizhou University of Chinese Medicine, Guiyang, 550001 China; 6 Department of Critical Care Medicine, General Hospital of Southern Theater Command of PLA, Southern Medical University, Guangzhou, 510010 China

**Keywords:** Glucocorticoid, COVID-19, Viral clearance

Dear Editor,

The coronavirus disease 2019 (COVID-19) outbreak has been a severe challenge worldwide. Accumulating evidence reveals that in COVID-19 patients, inflammatory cell infiltration and cytokine storm are key factors leading to acute lung injury and poor prognosis [1]. Glucocorticoid (GC) was one of the anti-inflammatory medications widely used in critically ill patients. Numerous clinical studies have reported the efficacy of GC in the treatment of coronavirus pneumonia; however, the use of GC in the treatment of critical COVID-19 cases is still controversial [2, 3]. The main concern is that GC treatment may delay the clearance of virus. The current cohort study aimed to determine whether GC therapy would prolong the duration of SARS-CoV-2 RNA shedding and SARS-CoV-2 clearance.

This cohort study analyzed clinical data from 684 adult patients with SARS-CoV-2 infections confirmed through RT-PCR on throat swab samples collected between January and March 2020 from two hospitals in Wuhan, China. All patients received standard treatment including antiviral and oxygen therapy, and symptomatic support. The demographic, laboratory data at admission and discharge, GC treatment, and prognosis of the patients were collected. During the treatment, the throat swab and/or sputum and/or lower respiratory tract samples from confirmed patients were collected and tested by RT-PCR every 2 to 3 days. For the severe and critical patients, the interval between two tests was 4 to 5 days. To avoid false negative results, only patients with three continuously negative tests were considered that they have viral RNA clearance. Therefore, for the patients with negative RT-PCR result, two more samples were collected in the following 2 days, respectively. Among the 684 cases, 202 (29.5%) cases had viral RNA clearance within 14 days after illness onset and 210 (30.7%) cases had viral RNA clearance between 14 and 28 days, and 272 (39.8%) cases had viral RNA clearance over 28 days. There were no differences on the age, gender, and underlying diseases between different groups. The degree of decrease in CD4 T cell and B cell counts on admission was related with the prolonged viral RNA clearance (Table 1).

**Table 1:** Demographics and laboratory data at admission of patients infected with COVID-19 according to the time to SARS-CoV-2 RNA clearance

	Overall (***N*** = 684)	≤ 14 days (***N*** = 202)	14–28 days (***N*** = 210)	> 28 days (***N*** = 272)	***p*** value
**Demographics, clinical characteristics**					
Age (years)	61.0 [49.0, 70.0]	62.0 [48.0, 71.0]	60.5 [49.3, 70.0]	61.5 [51.0, 70.3]	0.733
Gender (%)					0.841
Male	328 (48.0)	98 (48.5)	103 (49.3)	127 (46.7)	
Female	355 (52.0)	104 (51.5)	106 (50.7)	145 (53.3)	
Clinical type (%)					0.009
Mild	26 (3.9)	16 (8.0)	7 (3.4)	3 (1.1)	
General	464 (69.3)	140 (70.0)	141 (69.1)	183 (68.8)	
Severe	145 (21.6)	36 (18.0)	44 (21.6)	65 (24.4)	
Critical	35 (5.2)	8 (4.0)	12 (5.9)	15 (5.6)	
Hypertension (%)	235 (35.0)	67 (34.0)	70 (33.8)	98 (36.6)	0.778
CHD (%)	75 (11.2)	22 (11.2)	25 (12.1)	28 (10.4)	0.855
CRF (%)	16 (2.4)	3 (1.5)	7 (3.4)	6 (2.2)	0.467
DM (%)	111 (16.5)	30 (15.2)	36 (17.5)	45 (16.8)	0.824
COPD (%)	13 (1.9)	3 (1.5)	3 (1.4)	7 (2.6)	0.588
Cirrhosis (%)	3 (0.4)	0 (0.0)	1 (0.5)	2 (0.7)	0.492
Stroke (%)	45 (6.7)	14 (7.1)	14 (6.8)	17 (6.3)	0.943
Tumor (%)	23 (3.4)	5 (2.6)	5 (2.4)	13 (4.9)	0.257
**Inflammatory response, median (IQR)**					
CRP (mg/L)	7.39 [0.50, 28.25]	8.63 [1.04, 16.33]	5.79 [0.50, 28.80]	5.13 [0.50, 62.39]	0.968
IL-6 (pg/mL)	6.00 [2.25, 21.00]	6.00 [3.00, 19.50]	7.50 [2.00, 26.25]	6.00 [3.00, 20.50]	0.943
Fib (g/L)	4.82 [3.68, 11.90]	4.46 [3.58, 11.72]	5.50 [3.65, 11.98]	6.71 [4.08, 13.60]	0.19
WBC (10^9^/L)	5.59 [4.46, 7.06]	5.60 [4.47, 6.79]	5.51 [4.52, 7.12]	5.33 [4.38, 7.83]	0.962
Neutrophil (10^9^/L)	3.33 [2.44, 4.76]	3.33 [2.49, 4.51]	3.31 [2.42, 4.85]	3.67 [2.41, 5.83]	0.618
Monocyte (10^9^/L)	0.46 [0.34, 0.60]	0.45 [0.35, 0.59]	0.46 [0.34, 0.61]	0.47 [0.35, 0.58]	0.970
Lymphocyte (10^9^/L)	1.27 [0.88, 1.81]	1.29 [0.94, 1.86]	1.29 [0.95, 1.73]	1.00 [0.72, 1.67]	0.204
PLT (10^9^/L)	203 [158, 249]	209 [156, 267]	196 [158, 241]	189 [148, 244]	0.209
Hb (g/L)	121 [108, 133]	123 [109, 133]	121 [110, 131]	121 [103, 131]	0.609
CD3 (count/μL)	8905 [474, 1212]	916 [586, 1220]	705 [423, 1224]	497 [364, 684]	0.115
CD4 (count/μL)	478 [269, 672]	554 [319, 733]	324 [190, 588]	275 [154, 404]	0.048
CD8 (count/μL)	248 [150, 374]	291 [174, 400]	229 [142, 367]	167 [123, 211]	0.112
NK (count/μL)	167 [103, 259]	142 [91.0, 231]	206 [141, 332]	154 [91.8, 215]	0.115
B cell (count/μL)	175 [104, 281]	195 [131, 296]	187 [91.0, 279]	94.0 [72.8, 126]	0.030
**Organ function measurement, median (IQR)**					
ALT (U/L)	27.0 [18.0, 41.0]	26.0 [18.0, 40.5]	29.0 [19.0, 43.0]	27.0 [18.3, 38.3]	0.679
AST (U/L)	22.0 [16.0, 36.5]	20.0 [15.0, 36.0]	23.0 [17.0, 37.0]	23.0 [19.0, 39.0]	0.314
TBIL (μmol/L)	11.2 [8.40, 14.0]	11.3 [8.7, 14.0]	11.0 [8.30, 13.0]	11.1 [8.53, 15.8]	0.731
DBIL (μmol/L)	2.80 [2.10, 3.70]	2.70 [2.20, 3.60]	3.00 [1.92, 3.68]	2.90 [1.90, 4.40]	0.935
Creatinine (μmol/L)	61.0 [50.0, 75.0]	61.0 [50.0, 78.5]	60.0 [50.2, 74.7]	60.0 [50.2, 72.5]	0.694
BUN (mmol/L)	4.60 [3.60, 5.80]	4.40 [3.50, 5.80]	4.60 [3.70, 5.50]	4.50 [3.70, 5.80]	0.794
Lactate (mmol/L)	1.10 [1.00, 1.35]	1.00 [0.90, 1.30]	1.10 [1.00, 1.30]	1.10 [1.00, 1.60]	0.395
Glucose (mmol/L)	5.60 [5.00, 6.62]	5.55 [5.00, 6.32]	5.60 [5.03, 7.62]	5.30 [4.90, 6.95]	0.431
INR	1.10 [1.00, 1.20]	1.10 [1.00, 1.20]	1.10 [1.00, 1.20]	1.10 [1.10, 1.20]	0.075
CK (U/L)	66.5 [25.3, 110.5]	67.0 [24.0, 107.5]	62.0 [39.0, 135.0]	63.0 [22.5, 105.5]	0.530
BNP (pg/mL)	67.9 [28.0, 152.3]	67.9 [28.0, 243.0]	62.0 [27.0, 126.0]	97.0 [41.5, 128.8]	0.490
NT-proBNP (pg/mL)	745 [88.0, 1899]	109 [67.0, 2679]	1118 [769.5, 2237]	745 [382, 859]	0.532
**GC treatment (%)**	103 (15.1)	24 (11.9)	32 (15.2)	47 (17.3)	0.266
Methylprednisolone (%)	96 (14.0)	24 (11.9)	30 (14.3)	42 (15.4)	0.540
Dexamethasone (%)	12 (1.8)	2 (1.0)	3 (1.4)	7 (2.6)	0.392
Hydrocortisone (%)	1 (0.1)	0 (0.0)	0 (0.0)	1 (0.4)	0.468
**Outcome**					
Hospital stay (days)	25.0 [16.0, 38.0]	21.0 [14.0, 28.0]	24.0 [18.0, 32.0]	37.0 [21.0, 47.0]	< 0.001
Total course (days)	45.0 [33.0, 59.3]	30.0 [19.5, 39.5]	41.0 [32.0, 54.0]	57.0 [47.0, 67.0]	< 0.001
Outcome (%)					0.414
Survival	643 (96.8)	191 (97.0)	194 (95.6)	258 (97.7)	
Death	21 (3.2)	6 (3.0)	9 (4.4)	6 (2.3)	

Since GC therapy was usually employed in critically ill patients, we analyzed the effect of GC therapy separately for patients with different severity. Patients were diagnosed as mild type, general type, severe type, and critical type according to the *Chinese Recommendations for Diagnosis and Treatment of Novel Coronavirus (SARS-CoV-2) Infection (Trial 7th version)* [4]. For the mild and general type patients, 30 (6.1%) cases received GC treatment and 460 (93.1%) cases did not. For the severe and critical type patients, 72 (40%) cases were in the GC group and 108 (60%) cases were in the non-GC group (Table 1). In this study, methylprednisolone was the most used glucocorticoid (Table 1) in a dose of 1–2 mg/(kg·day) for 3 to 5 days according to the disease severity [4]. The results show that GC therapy increased hospital stay days but had no effect on the virus clearance time (Table 2). For the severe and critical patients, the median viral RNA clearance time in the GC group was 26 days (IQR 17–42 days), while the viral RNA clearance time in the non-GC group was 25.5 days (IQR 13–39 days). In addition, the GC treatment had no effect on the peripheral lymphocyte counts, including CD4 T cells, CD8 T cells, NK cells, and B cells (Table 2).

**Table 2:** Effect of glucocorticoid on the outcome and inflammatory response on discharge of COVID-19 patients

Variables	Mild and general group			Severe and critical group		
	Non-GC group	GC group	***p*** value	Non-GC group	GC group	***p*** value
*N*	460	30		108	72	
Hospital stay (days)	23.00 [16.00, 33.00]	32.50 [23.25, 38.00]	0.002	29.00 [17.00, 44.00]	40.50 [31.75, 52.50]	< 0.001
Viral RNA clearance (days)	22.00 [11.00, 35.00]	23.50 [14.00, 34.25]	0.737	25.50 [13.00, 39.00]	26.00 [17.00, 42.00]	0.471
Total course (days)	43.00 [29.00, 57.00]	41.00 [31.50, 47.75]	0.816	49.00 [37.50, 63.50]	49.00 [43.00, 63.00]	0.341
Outcome (%)			1			0.555
Survival	446 (99.8)	30 (100.0)		92 (90.2)	62 (86.1)	
Death	1 (0.2)	0 (0.0)		10 (9.8)	10 (13.9)	
**Inflammatory response, median (IQR)**						
CRP (mg/L)	2.93 [0.90, 10.00]	4.15 [2.04, 10.50]	0.378	3.19 [0.77, 21.20]	4.59 [1.16, 13.15]	0.457
IL-6 (pg/mL)	3.00 [2.00, 7.00]	5.00 [2.00, 12.50]	0.253	16.00 [4.75, 36.00]	9.50 [7.25, 32.00]	0.986
Fib (g/L)	3.58 [2.78, 4.84]	3.96 [3.58, 11.43]	0.227	3.53 [2.84, 14.39]	3.90 [3.60, 10.90]	0.275
WBC (10^9^/L)	5.55 [4.40, 6.55]	5.20 [4.25, 6.08]	0.568	5.93 [5.00, 7.50]	6.80 [4.90, 8.55]	0.485
Neutrophil (10^9^/L)	3.17 [2.49, 4.04]	3.05 [2.25, 3.98]	0.641	3.76 [2.85, 5.20]	4.79 [3.09, 6.80]	0.178
Monocyte (10^9^/L)	0.46 [0.36, 0.57]	0.49 [0.41, 0.66]	0.177	0.43 [0.35, 0.66]	0.51 [0.40, 0.63]	0.311
Lymphocyte (10^9^/L)	1.56 [1.25, 1.91]	1.47 [1.23, 1.88]	0.801	1.21 [0.97, 1.79]	1.10 [0.85, 1.55]	0.207
PLT (10^9^/L)	209.0 [173.0, 258.0]	212.0 [183.8, 266.5]	0.923	212.0 [151.0, 236.0]	183.0 [144.0, 250.0]	0.615
Hb (g/L)	128.5 [117.25, 138.8]	133.0 [127.0, 137.0]	0.667	117.5 [102.5, 129.0]	119.0 [100.0, 125.0]	0.669
CD3 (count/μL)	1144 [861, 1293]	1002 [793, 1282]	0.791	509 [162.0, 1026]	683.5 [478.8, 1112]	0.508
CD4 (count/μL)	625.0 [514.0, 831.5]	498.0 [385.0, 801.0]	0.442	306.0 [65.3, 585.5]	338.5 [257.5, 547.0]	0.449
CD8 (count/μL)	357.0 [246.0, 454.0]	364.0 [248.5, 478.5]	0.845	133.0 [72.3, 341.5]	257.0 [190.3, 389.3]	0.257
NK (count/μL)	201.0 [128.0, 271.5]	90.0 [55.0, 132.0]	0.008	52.5 [40.0, 124.00]	93.50 [62.5, 117.3]	0.257
B cell (count/μL)	171.0 [132.5, 265.0]	248.0 [146.0, 328.5]	0.493	68.5 [58.5, 126.8]	115.0 [94.3, 156.0]	0.299

The current multicenter cohort study demonstrates that GC therapy does not change viral clearance and peripheral lymphocyte counts in COVID-19 patients. However, well-designed and large-scale randomized controlled trials are needed to further confirm the results derived from this observational study.

## Data Availability

The datasets used and/or analyzed during the current study are available from the corresponding author on reasonable request.
